# Tumor-Agnostic Therapy—The Final Step Forward in the Cure for Human Neoplasms?

**DOI:** 10.3390/cells13121071

**Published:** 2024-06-20

**Authors:** Mohamed Mahmoud El-Sayed, Julia Raffaella Bianco, YiJing Li, Zsolt Fabian

**Affiliations:** School of Medicine and Dentistry, Faculty of Clinical and Biomedical Sciences, University of Central Lancashire, Preston PR1 2HE, UK; mmelsayed@uclan.ac.uk (M.M.E.-S.); jrbianco@uclan.ac.uk (J.R.B.); yjli4@uclan.ac.uk (Y.L.)

**Keywords:** tumor-agnostic philosophy, pan-cancer mutations, biomarkers, next-generation sequencing

## Abstract

Cancer accounted for 10 million deaths in 2020, nearly one in every six deaths annually. Despite advancements, the contemporary clinical management of human neoplasms faces a number of challenges. Surgical removal of tumor tissues is often not possible technically, while radiation and chemotherapy pose the risk of damaging healthy cells, tissues, and organs, presenting complex clinical challenges. These require a paradigm shift in developing new therapeutic modalities moving towards a more personalized and targeted approach. The tumor-agnostic philosophy, one of these new modalities, focuses on characteristic molecular signatures of transformed cells independently of their traditional histopathological classification. These include commonly occurring DNA aberrations in cancer cells, shared metabolic features of their homeostasis or immune evasion measures of the tumor tissues. The first dedicated, FDA-approved tumor-agnostic agent’s profound progression-free survival of 78% in mismatch repair-deficient colorectal cancer paved the way for the accelerated FDA approvals of novel tumor-agnostic therapeutic compounds. Here, we review the historical background, current status, and future perspectives of this new era of clinical oncology.

## 1. Introduction

Cancer is among the leading causes of mortality in developed countries due to the high rate of morbidity and limited efficiency of contemporary therapeutic modalities. Indeed, lung cancer, for instance, accounts for an estimated 350 fatalities daily, making it the leading cause of cancer-related mortality across both sexes in 2022. From 2001 to 2017, an increase from 19% to 31% in 3-year survival outcome was observed. During this period, the median survival increased from 8 to 13 months [1]. Despite conventional chemotherapy being the primary treatment for non-small-cell lung cancer, the success rate was less than 5% [2].

Over the course of several decades, cancer management has predominantly relied on scrutinizing tumor tissue at the cellular and supracellular levels [3]. This has led to a site-specific approach in managing tumors, where guidelines are tailored, primarily, on the basis of their location within the human body. Accordingly, cancer research, clinical trials, and the development of anticancer drugs have, so far, hinged on histopathological classifications rather than addressing genetic alterations, highlighting the inherent limitations of these treatment regimes. Moreover, the lack of specificity of conventional therapies has been associated with adverse side effects and toxicity, while they may become ineffective over time as cancer cells develop drug resistance [4,5,6]. The limitations of conventional approaches urged a paradigm shift in developing new anticancer therapies that considers the unique molecular characteristics of cancer cells.

Accordingly, the landscape of cancer treatment has undergone a remarkable transformation with the rapid evolution of clinical genomic testing and simultaneous strides in precision cancer therapies. This has given rise to genomically targeted therapies and immune-boosting agents that represent a groundbreaking shift in cancer therapeutics.

Indeed, the repertoire of targeted therapies, designed to address specific molecular alterations, has witnessed an exponential surge in recent years [7]. The introduction of immunotherapy has propelled cancer treatment to a new dimension by harnessing the body’s own defenses to combat the disease [8]. Consequently, we find ourselves moving beyond the era of site-specific cancer management, witnessing the emergence of tissue-agnostic biomarkers spanning diverse cancer histologies. This shift has been primarily fueled by actionable alterations identified through next-generation sequencing and the development of drugs capable of precisely targeting these alterations.

While initially considered speculative, recent years have provided compelling evidence supporting the efficacy of biomarker-driven, tissue-agnostic therapeutics. Regulatory approvals have played a crucial role in validating this paradigm shift [7,9].

## 2. Tumor-Agnostic Philosophy

### 2.1. Advances in Understanding the Molecular Events of Tumor Development

**Figure 1:**
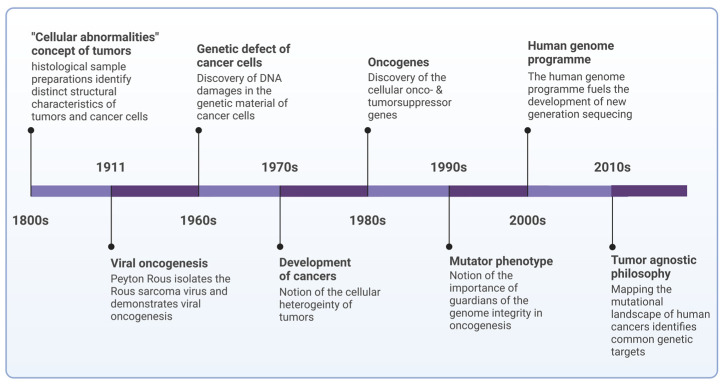
Historical timeline of the development of our understanding of human neoplasms.

Advancements in the preparation of histological samples and staining techniques for biological materials enabled researchers to offer a more intricate understanding of tumor structures. In the early 1800s, several scholars observed that neoplastic tumors consisted of cells with irregular shapes. This insight was initially articulated by Schleiden and Müller in 1838, and later corroborated by Schwann in 1839 [10]. They identified distinctive characteristics of neoplastic tumors, such as an increased rate of cell division, abnormal mitotic activity, enlarged cell nuclei, and a loss of cellular differentiation. Moreover, they noted additional tumor features like the surrounding stroma, blood vessels, and areas of necrosis, particularly within the cores of tumors. By the late 1800s, an increasing number of researchers leaned towards Virchow’s theory, that the root cause of the disease lay in cellular abnormalities (Figure 1) [11].

During the 1970s, it became evident that the cells composing tumor masses display remarkable heterogeneity. They exhibit variations, such as differing resistance to drugs or sensitivity to signals prompting programmed cell death. Furthermore, over time, these cells become increasingly aggressive and acquire new capabilities, such as the ability to metastasize. While the “disease of genes” model of cancer recognized the role of mutations in initiating the disease, it failed to provide a comprehensive explanation for this progression. Consequently, it was inferred that there must exist a mechanism facilitating the emergence and accumulation of successive alterations.

The concept that viruses contribute to cancer development traces back to the research published by Peyton Rous in 1911 [12]. Rous identified a filterable agent, termed Rous sarcoma virus (RSV), in cell extracts from a chicken tumor capable of inducing tumors in healthy chickens. This groundbreaking discovery of a retrovirus laid the foundation for tumor virology, highlighting that certain cancers may have an infectious origin [13]. In the 1960s and 1970s, the first tumor viruses affecting humans were identified. The Epstein–Barr virus (EBV), also known as human herpes virus 4 (HHV-4), was first visualized in cells cultured from Burkitt’s lymphoma using electron microscopy, marking the inception of human tumor virology [14]. Subsequent immunofluorescence assays revealed heightened immune responses to EBV antigens in individuals with Burkitt’s lymphoma or nasopharyngeal carcinoma, and biopsy samples from these cancers exhibited the presence of EBV DNA [15].

By the 1960s, it became evident that cancer development is closely linked to genetic errors, which are passed on to descendant cells during subsequent divisions. This led to a shift in the interpretation of carcinogenesis from a cellular level to a molecular one, focusing on genetic abnormalities rather than cellular dysfunction [16]. The 1980s witnessed a surge in research focused on genes implicated in carcinogenesis. It became apparent that several genes identified in oncoviruses shared identical sequences with their cellular counterparts (e.g., SRC, initially discovered in avian DNA), eventually leading to the identification of cellular oncogenes [17].

In the early 1980s, orthologs of viral *RAS* oncogenes with point mutations were identified in DNA fragments from human cancer cells, marking a significant milestone in molecular oncology research [18,19]. This was followed by the discovery of cellular counterparts of further viral oncogenes, tumor drivers initially identified in retroviruses, including *MYC*, *RAF*, *ERBB1* (encoding Epidermal growth factor receptor [EGFR]), *AKT*, and *SIS* (encoding subunit B of the platelet-derived growth factor [PDGF]). Subsequent research revealed their key involvement in growth signaling pathways [20].

Research into chromosome abnormalities in cancer cells has revealed a striking finding: tumor cells may harbor an extensive array of mutations, possibly numbering in the thousands [21,22]. This substantial quantity prompted Loeb et al. to formulate the “mutator phenotype hypothesis,” suggesting that the conventional mutation rate in healthy cells falls short of explaining the abundance of mutations observed in cancer cells [23]. Consequently, mutations occurring in genes governing DNA synthesis fidelity or DNA repair (caretaker genes), as well as those encoding proteins involved in cell cycle regulation or apoptosis (gatekeeper genes), could significantly elevate the baseline mutation rate, potentially elucidating the multiple mutations found in tumor cells. The considerable surge in cancer risk as individuals age coincides with a shift from hematopoietic tumors, which tend to occur earlier in life, to epithelial carcinomas [24].

Genes implicated in the process of carcinogenesis are typically divided into three main categories: proto-oncogenes, tumor suppressor genes, and mutator genes. Proto-oncogenes are responsible for encoding proteins that stimulate cell division. When these genes undergo mutations, the resulting proteins can be overactivated, leading to uncontrolled cell proliferation. Conversely, tumor suppressor genes produce proteins that physiologically inhibit cell division and promote cell survival [25]. Mutator genes, on the other hand, are responsible for maintaining genome integrity, so upon their loss-of-function mutations, they induce chromosomal instability, which can result in mutations in proto-oncogenes and tumor suppressor genes. Additionally, mutator genes can lead to other mutations that confer a selective advantage for certain tumor cell clones to survive in their environment [26].

Current understanding suggests that the initiation of carcinogenesis typically requires gain-of-function mutations of proto-oncogenes and loss-of-function mutations of the tumor suppressor ones. Mutations in genes encoding proteins involved in apoptosis, DNA damage detection, and repair also play significant roles in oncogenesis. Cancer cells undergo a process of clonal selection, wherein the initial cells carry a variety of mutations, leading to diversity among their offspring [27]. While most genomic alterations have either neutral or detrimental effects on the cell phenotype, a select few confer a survival advantage. These advantageous clones proliferate and eventually dominate the tumor’s growth [26]. As tumor progression ensues, features that enhance cellular survival accumulate, such as genome instability, which increases the likelihood of additional mutations. Through clonal evolution, the most adapt clones emerge from the heterogeneous population of cancer cells to thrive within the tumor environment [28]. It is believed that cancer usually arises from multiple mutations in tumor-driver genes, with the accumulation of mutations being more critical than their specific sequence of appearance [29].

### 2.2. Comparative Studies on the Molecular/Genetic Landscape of Individual Tumors

Advancements in our understanding of the carcinogenesis along with the development of high-throughput genomics inevitably led to the call for comprehensive investigations of the genetic landscapes of diverse neoplasms for mapping their molecular constellations [30]. Indeed, The Cancer Genome Atlas (TCGA) has conducted several pan-cancer genome studies, aiming to comprehensively analyze thousands of cancer genomes across multiple cancer types [31,32,33,34,35]. These studies have led to the extraction of 21 characteristic mutations from 30 types of cancers, providing a comprehensive landscape and dictionary of mutational signatures in major cancers [31]. Another systematic analysis of 3281 tumors from 12 cancer types to investigate the underlying mechanisms of cancer initiation and progression identified 127 significantly mutated genes involved in diverse cellular processes, including *TP53*, *PTEN*, *APC*, *KRAS*, and *RB* [35]. Identified genes were broadly classified into 20 categories, such as transcription factors/regulators, histone modifiers, transcription regulators, metabolic pathway elements, and genome integrity maintenance mediators [36].

*TP53* emerged as the most frequently mutated gene in the pan-cancer cohort (42% of samples), with mutations being particularly prevalent in serous ovarian (95%) and serous endometrial carcinomas (89%). *PIK3CA* followed as the second most commonly mutated gene, observed frequently across most cancer types except ovarian serous carcinoma (OV), kidney renal clear cell carcinoma (KIRC), lung adenocarcinoma (LUAD), and acute myeloid leukemia (AML). Mutations in *PIK3CA* were notably enriched in luminal subtype tumors, notably in uterine corpus endometrial carcinoma (UCEC) (52%) and breast adenocarcinoma (BRCA) (33.6%). Interestingly, tumors lacking *PIK3CA* mutations often harbored mutations in *PIK3R1*, with significant occurrences in UCEC (31%) and glioblastoma multiforme (GBM) (11%) [35].

Chromatin remodeling genes displayed mutations across many cancer types, with histone-lysine N-methyltransferase genes (*MLL2*, *MLL3*, and *MLL4*) clustering notably in bladder, lung, and endometrial cancers. Additionally, mutations in *ARID1A* were frequent in bladder urothelial carcinoma (BLCA), UCEC, LUAD, and lung squamous cell carcinoma (LUSC), while *ARID5B* mutations were prevalent in UCEC (10%). Mutually exclusive *KRAS* and *NRAS* mutations were identified, with recurrent activating mutations observed commonly in colon and rectal carcinoma (COAD/READ) (30%, 5%, and 5% for *KRAS* (Gly 12), *KRAS* (Gly 13), and *NRAS* (Gln 61), respectively) [35].

Further intriguing insights have been uncovered by another large-scale survey (MSK-IMPACT) using prospective sequencing on tumors from over 10,000 cancer patients representing a diverse spectrum of solid tumor types [30]. The findings revealed that certain genes, previously deemed significant in TCGA studies, exhibited even higher mutation frequencies within the MSK-IMPACT dataset. Particularly notable was *TP53*, which displayed significantly elevated mutation rates across four tumor types (prostate cancer, kidney chromophobe carcinoma, glioblastoma, and gastric cancer) compared to TCGA. In prostate cancer alone, *TP53* mutations were over four times more prevalent in MSK-IMPACT (29%) than in TCGA (7%), consistent with prior observations linking *TP53* mutations to more aggressive disease [37].

*TP53* emerged as the most frequently altered gene in the MSK-IMPACT dataset, affecting 41% of patients. Its mutations were notably prevalent in high-grade serous ovarian cancer (98%), esophageal adenocarcinoma (89%), and small-cell lung cancer (85%), often leading to functional inactivation through truncation or splicing disruption. Across 43 of the 62 primary tumor types studied, *TP53* alterations were present in over 10% of cases.

Following *TP53*, *KRAS* ranked as the second most frequently altered gene (15% of patients), with mutations predominantly observed in pancreatic adenocarcinoma (90%) and colon adenocarcinoma (44%). The G12 codon of *KRAS* stood out as the most frequently altered among the sequenced tumors, accounting for 80% of all *KRAS* mutations and 12% of individuals sequenced. Other commonly mutated loci included *PIK3CAH1047*, *PIK3CAE545*, and *BRAFV600*, each occurring in over 20 principal tumor types, indicating positive selection for these variants across diverse cancer lineages [38].

While some genes, like *TP53* and *PIK3CA*, displayed consistent mutation rates across multiple tumor types, others such as *VHL*, *APC*, and *IDH1* showed high mutation rates limited to only a few types of neoplasms. Certain gene fusions were also uniquely associated with particular cancer lineages, such as *TMPRSS2-ERG* in prostate cancer, *EWSR1-FLI1* in Ewing sarcoma, and *DNAJB1-PRKACA* in fibrolamellar hepatocellular carcinoma. However, excluding tumors with unusually high mutation rates, 97% of genes in the 410-gene panel showed mutations in at least five major tumor types, highlighting the potential benefits of comprehensive mutation profiling regardless of cancer lineage.

In addition to targeting the coding sequences of cancer-associated genes, MSK-IMPACT also captured the promoter region of *TERT*. Mutations occurring at two frequently recurring sites in the *TERT* promoter have been observed to generate new consensus binding sites for ETS family transcription factors. This leads to increased telomerase expression and decreased cell death. TERT promoter mutations were most commonly found in bladder cancer (70%), glioma (67%), thyroid cancer (60%), and melanoma (49%), particularly cutaneous melanoma. The researchers consistently noted a trend towards shorter survival among individuals with altered TERT promoters [39,40].

### 2.3. Advances in Molecular Characterization of Patient Tumors

**Figure 2:**
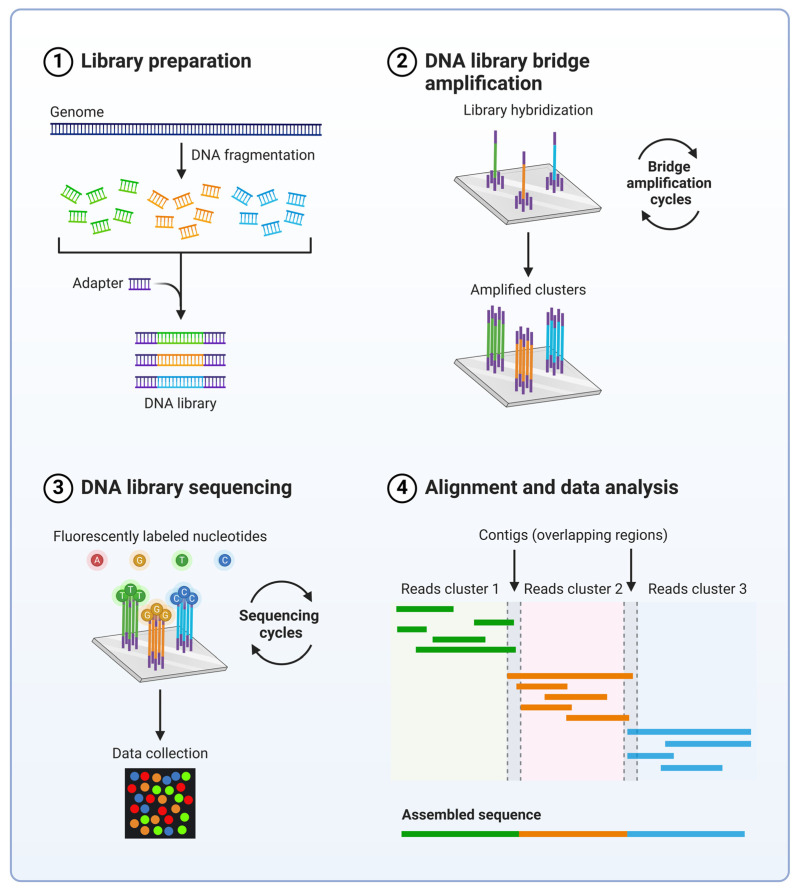
Schematic overview of the principle of the next-generation sequencing methodology. NGS uses libraries of short segments of DNA or RNA tagged by adapter sequences for bidirectional bridge amplification. Sequencing is accomplished using the amplified fragments using fluorescently labeled nucleotides of which emission wavelengths and intensity are continuously recorded upon the simultaneous synthesis along millions of templates. This leads to the read of hundreds to thousands of fragment clusters at the same time. Sequence information is finally lined up to reference genome sequences.

Advances in our understanding of the genetic mechanisms of tumor formation by large-scale genomic surveys for cancer-driver mutations, like TCGA and the International Cancer Genome Consortium (ICGC), paved the way for growing demands for high-throughput diagnostic tools for screening characterized molecular hallmarks of cancer in patient samples [41,42]. This fueled the implementation of novel technologies like next-generation sequencing (NGS) in the clinical practice of tumor diagnostics (Figure 2). NGS allows for the sequencing of longer DNA sections faster than that of the current canonical sequencing technologies for a fraction of the cost. Using short segments of DNA or RNA, NGS uses libraries of these fragments tagged by adapter sequences that allows for efficient bidirectional amplification of all nucleotide fragments using standard primers generating both forward and reverse amplicons [43]. The process known as bridge amplification results in parallel amplification of each fragment of the sample. Sequencing is accomplished using the amplified fragments as template strands and polymerases that incorporate fluorescently labeled nucleotides to the growing strand. Fluorescently labeled nucleotides emit their characteristic signals upon incorporation. Their emission wavelengths and intensity are recorded to determine what nucleotide has been added to the growing strand. Synthesis takes place simultaneously along millions of templates in a common platform, and the emitted signal pattern is continuously recorded. This leads to the read of hundreds to thousands of fragment clusters at the same time, making the technology much more efficient than previous sequencing methods. Sequence information of the fragments finally lines up to reference genome sequences by dedicated algorithms to create the final sequence of the nucleotide sample tested (Figure 2) [43].

NGS not only allows for broad mutation detection with faster speeds, allowing for early detection of neoplasms, but requires less patient sample material compared to other detection options [44]. The high-throughput nature of the NGS technology combined with the comprehensive catalog of cancer-driver genetic alterations allowed the innovation of NGS-based diagnostic panels that provide concise and affordable screening for cancer biomarkers in everyday clinical practice.

Indeed, over the past few years, several NGS-based cancer biomarker screening kits have been developed and introduced successfully to the hospital laboratory repertoire. One of the first FDA-approved functional screening panels (MSK-IMPACT) utilizing hybridization capture technology and next-generation sequencing was developed by the Memorial Sloan Kettering Cancer Center. This innovative panel has the power of not only screening for pathognomonic mutations within protein-coding regions but also detects copy number alterations, selected promoter mutations, and structural rearrangements across 410 onco- and tumor suppressor genes [45]. Without being limited to a given cancer type, the MSK-IMPACT panel has been successfully used in clinical settings for both aiding in patient’s enrollment to clinical trials and defining targeted drug therapies [45]. Since then, oncopanels have been developed further with different specifics and have become widely used in the current clinical practice (Table 1). Although NGS tends to be the most common approach to biomarker identification, it does not provide information on the biomarker status of target cells at the protein level. New advances in immunohistochemistry (IHC) can be used as another biomarker screening tool. Large-scale immunohistochemistry screening on tissue microarrays and tissue slice arrays has been proposed as a lower-cost and more efficient option with the aim to complement targeted therapy efforts [46].

Programmed death ligand 1 (PD-L1) binds to programmed death receptor 1 (PD-1) on lymphocytes, causing the lymphocytes to be inactivated. Some cancer cells have been seen to have an increase in PD-L1, resulting in host immune cells to be rendered inactive against cancer cells. PD-L1 immunohistochemistry assays are now in use to identify PD-L1-positive cancers. Currently, there are four main stains being worked on, 22C3, 28-8, SP142, and SP263, which differ in the antibodies used (Table 2) [52,53]. PD1 IHC has been of keen interest in triple-negative breast cancer and lung cancer [52,53,54].

It has been made very evident that the use of biomarkers is of great importance when it comes to early diagnosis and treatment options; therefore, there is a continuous need to make the discovery and identification of such biomarkers more efficient. In the detection of colorectal cancer (CRC), there is a very large influx of patient samples needed to be tested due to all individuals aged 50–75 in North America needing to be screened. Due to the large volume, more efficient and more cost-effective methods are advantageous. Currently, the common form of diagnosing CRC is through fecal occult blood testing, which tends to have a high false positive and false negative rate, or colonoscopies, which are quite invasive. High-throughput proteomic platforms allow for the screening of protein biomarkers. In high-throughput proteomic platforms, antibody mixtures are used to identify antigens on the protein that has been extracted from the biopsy. This method can be used in the detection of CRC via the use of aptamer-based screening. Stool is used as the sample and the results are verified using ELISA [59].

An emerging technique for the detection of biomarkers is electrical biosensors. Electrical biosensors allow you to identify a protein biomarker using molecular imprinting polymers (MIPs), which allow you to create a synthetic receptor for the biomarker you are looking for. The formation of these synthetic receptors has been shown to have more rapid and accurate identification of biomarkers [60]. Electrical biosensors have been showing fast and accurate readings at low detection levels, making them a very useful potential product in everyday care [61]. Electrical biosensors have already been used for both detecting well-known cancer biomarkers such as CEA and searching for new biomarkers like STEAP1, opening new frontiers in cancer diagnosis and management [60,61]. This technology can be very useful in the investigation of tumor-agnostic targets as well as opening the door to new targets through the ability to create a synthetic receptor, as natural receptors such as enzymes and antibodies can be high in cost as well as requiring strict conditions to function [60].

### 2.4. Common Molecular Characteristics of Histopathologically Diverse Tumors

**Figure 3:**
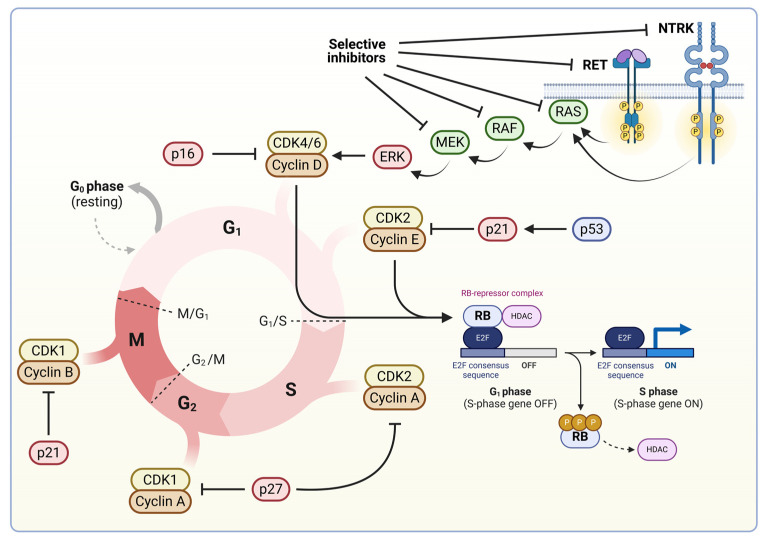
Schematic overview of the roles of characteristic tumor-agnostic biomarkers in cell cycle control.

Using rapidly evolving contemporary high-throughput technologies, the tumor-agnostic approach became a clinical reality over recent years. By screening for characterized common tumor drivers, they provide information in continuously increasing details of the genetic constellation of individual tumors. These data shed light on biomarkers frequently occur in tumors that, formerly, were characterized as unrelated. Cell surface receptors are prominent examples of such tumor-agnostic markers (Figure 3).

Indeed, neurotrophic tyrosine kinase receptors (NTRKs), for instance, have been found in several adult and pediatric malignances, including infantile fibrosarcoma, congenital mesoblastic nephroma, and papillary thyroid cancer [Table 1] [62]. In non-transformed tissues, NTRKs are involved in neuronal growth, differentiation, and survival. In transformed cells, however, NTRK encoding genes are often found to be fused with other genes, leading to their own constitutive upregulation and the consequent activation of their downstream effectors, including elements of the phospholipase Cγ, mitogen-activated protein kinase (MAPK), and phosphatidylinositol 3 phosphate kinase signaling pathways [63,64].

The latter ones are particularly common targets for mutated cell surface receptors even beyond NTRKs. Anaplastic lymphoma kinase (ALK), a tyrosine kinase receptor mainly expressed in the brain, testis, and small intestines, also signals through the MAPK and PI3K pathways, regulating cell survival and proliferation [65]. *ALK* also seems to be prone to chromosomal rearrangements, resulting in fusion genes with recurring fusion partners like the nucleophosmin 1 gene (*NPM1*) or EMAP like 4 gene (*EML4*) [66,67]. *NPM1* produces chaperone proteins as wells as proteins that protect tumor suppressor protein ARF from being degraded by sequestering ARK into the nucleolus, while *EML4* is suspected to produce proteins involved in microtubule formation [68,69]. In these chimeras with its 5′ region being lost, the 3′ end of *ALK* usually remains intact, preserving its kinase and allowing ALK to be active without a ligand present, giving rise to constitutively active ALK activities. As a tumor-agnostic target, *ALK* rearrangements have been found in NSCLC, anaplastic large cell lymphoma, and breast cancer, among others [62,70,71].

Another receptor tyrosine kinase, ERBB2, also known as HER2, has also emerged as a powerful tumor-agnostic molecular signature. Although traditionally it has been known for its assignment with breast cancer, *ERBB2* overexpression, usually due to in-frame insertions and amplifications, has been identified in CRC and NSCLC alike [71,72,73]. ERBB2 is a member of the epidermal growth factor receptor family, primarily signaling through the MAPK cascade and phosphatidylinositol pathways, orchestrating the expression of genes involved in the regulation of cell cycle progression and survival [74]. Besides ERBB2, the RET receptor tyrosine kinase plays a similar role among the cell surface cohort of tumor-agnostic biomarkers. *RET* plays a central role in early human development, but in adult tissues, its functions are unclear [75]. *RET* fusions with other genes, commonly *KIF5B*, cause RET receptor tyrosine kinase to be constitutively active and have been frequently detected in NSCLC and papillary thyroid neoplasms [76]. In accordance with the tumor-agnostic philosophy, however, mutated *RET* variants were also found in neoplasms of breast tissue, widening the diagnostic and treatment repertoire for HER2-negative and -positive breast cancers which are resistant to current traditional treatment [77,78].

Abnormal signaling from cell surface receptors eventually converges on conserved signaling pathways, like the MAPK one, that play central roles in the regulation of the cell cycle. Thus, it is not surprising that rate-limiting elements of these signal transduction machineries are also commonly found to be mutated in histologically diverse neoplasms and, therefore, could serve as tumor-agnostic biomarkers. One of these proven candidates is *KRAS*, which produces a protein of the RAS family. KRAS undergoes a conformational change when bound to GTP that allows its interaction with GTPase-activating proteins (GAPs), resulting in its endogenous GTPase activity being amplified [79,80]. Several solid tumors, including NSCLC and colorectal cancers, are associated with *KRAS* mutations, of which the most common one is G12C missense mutations [81,82,83]. G12C produces a KRAS that is more favorably bound to GTP, rendering KRAS constitutively active [84]. Constitutive activation of KRAS will result in the upregulation of downstream components of the MAPK pathway, leading to the dysregulation of the cell cycle [84]. *BRAF*, a serine/threonine protein kinase that functions as a proximal effector of the MAPK pathway downstream of RAS, is also commonly mutated in histologically unrelated human tumors, making BRAF another tumor-agnostic target [85]. Indeed, although mutated *BRAF* has, traditionally, been associated with melanoma, it has also been identified in gliomas, NSCLC, colorectal tumors, head and neck neoplasms, and hepatocellular carcinomas [71,85,86]. *BRAF*’s most common cancer-causing missense mutation, V600E, renders BRAF to be constitutively active as a monomer, leading to abnormal firing of the critical intracellular machinery that governs cell cycle progression [86].

Tumor suppressor genes also play a central role in the regulation of cell division, so it is not surprising that their mutations are seen in almost all human neoplasms [87]. Indeed, *TP53*, which encodes for p53, a protein involved in cell cycle arrest and apoptosis, is one of the most commonly mutated genes in human neoplasms, predicting it to be an ideal tumor-agnostic target [35]. However, extensive research on *TP53* revealed more than 1000 diverse mutations, some of which are dominant negative ones that make the mutant variant interfere with wildtype p53, while other mutations are rather loss-of-function ones [88]. The extensive variety of mutations plays into the difficulty in using *TP53* as a direct tumor-agnostic target. The cell cycle regulator retinoblastoma protein (Rb), which blocks cell cycle progression via E2F, is also commonly mutated in diverse human tumors from the originally associated pediatric retinoblastoma to breast cancer or osteosarcoma [89,90]. E2F is a regulatory transcription factor that mediates cell cycle progression by controlling the expression of genes like cyclins [91]. Upon the CDK4/6-mediated phosphorylation of Rb, E2F is released, and the cell prepares for cell division by entering the S phase. Mutated *RB* fails to block E2F, allowing cells to progress through the cell cycle [91]. Interestingly, *Rb*, similar to *TP53*, also shows a high degree of mutational mosaicism, at least in part, due to the considerable quantity of CpG islands within the gene, making its direct pharmacological targeting particularly challenging [92,93]. Thus, current research is moving towards marking additional elements of the p53 and Rb pathways, like the negative p53 regulator MDM2 or the CDK4/6 that inhibits Rb, for potential use as tumor-agnostic biomarkers [94,95,96].

To prevent the accumulation of mutations in genes including the proto-onco- and tumor suppressor ones, maintenance of the genome integrity is critical. Thus, mutations of the mutator genes, including both caretaker genes, which are involved in DNA repair and genome stability, and gatekeeper genes, which are involved in apoptosis, are also widespread in transformed cells, making them potential tumor-agnostic targets [97]. Indeed, germline and acquired mutations in the emblematic member of the caretaker genes, breast cancer gene 1 and 2 (*BRCA1*/2), are ordinarily associated with breast cancer but have been documented in ovarian and prostate cancers as well [98,99,100]. The mutational pattern of *BRCA1/2*, however, poses challenges. Hereditary *BRCA1/2* mutations vary amongst ethnic groups studied, and there is no singular mutation associated with acquired *BRCA1*/2. In the Japanese population, L63X and Q934X of *BRCA1* are the most common mutations, while in the Ashkenazi Jewish population, 5382insC or 185delAG in *BRCA1* and 6174delT in *BRCA2* are the most prevalent [101,102]. *BRCA1/2* encodes for proteins involved in the repair of DNA double-strand breaks [100]. Thus, *BRCA1/2* mutations, along with mutations in other mutator genes like *ATM* and *CHEK2*, have been exploited as tumor-agnostic markers, but in a unique application [87]. *ATM* produces a protein kinase that, when activated, allows for the downstream signals of DNA repair activity to begin, including the activation of *BRCA1/2* and *CHEK2* [103]. *CHEK2* produces a protein which is rapidly phosphorylated upon DNA damage and prevents the cell from entering mitosis [104]. CHEK2 is also responsible for the activation of BRCA1 and BRCA2 [104]. Inhibition of the poly(adenosine diphosphate [ADP]–ribose) polymerases (PARPs), which are involved in single-strand break repairs, in neoplasms with *BRCA1/2*, *ATM* and *CHEK2* mutations causes single- and double-strand breaks at the replication forks which will, eventually, lead to the disassembly of the replication fork and, consequently, cell cycle arrest [87,105,106,107].

### 2.5. Molecular Signatures of the Tumor Microenvironment for Tumor-Agnostic Targets

Besides the intracellular ones, breakthrough discoveries in tumor immunology shed light on molecular signatures that can serve as tumor-agnostic targets at supracellular levels as well. Indeed, with the rapidly growing attention to the role of the immune system in the development of neoplasms, molecules that are critical in the interaction between the immunocompetent cells and the tumor microenvironment emerged as primary targets for contemporary tumor-agnostic treatment regimes.

The tumor microenvironment (TME) is the complex and dynamic cellular environment where a tumor develops. It includes various immune cells such as T and B lymphocytes, tumor-associated macrophages, dendritic cells, natural killer cells, neutrophils, and myeloid-derived suppressor cells; stromal cells like cancer-associated fibroblasts, pericytes, and mesenchymal stromal cells; the extracellular matrix; and secreted molecules such as growth factors, cytokines, chemokines, and extracellular vesicles [108]. Additionally, it encompasses the blood and lymphatic vascular networks. These elements are interconnected and interact with the heterogeneous cancer cells. The TME significantly contributes to the acquisition and maintenance of cancer hallmarks, such as sustaining proliferative signaling, evading cell death, promoting angiogenesis, enabling invasion and metastasis, triggering tumor-promoting inflammation, and avoiding immune destruction [109]. Due to the pivotal role of the TME in tumor progression and its influence on the effectiveness of cancer treatments, strategies to target the TME have greatly expanded in recent years. These approaches mainly focus on targeting TAMs, DCs, T cells, tumor vasculature, extracellular matrix, and cancer-associated fibroblasts.

Within the TME, tumor-associated macrophages (TAMs) can quickly respond to local stimuli such as cytokines or therapeutic interventions, adopting a range of phenotypes from proinflammatory to anti-inflammatory states [110,111]. This plasticity is influenced by factors such as the stage of the disease, the affected tissue, and the host microbiota, collectively determining whether TAMs inhibit or promote tumorigenesis [112,113]. Targeting macrophages in therapy offers dual benefits: it can block TAMs from directly aiding cancer cell survival and can enhance the cross-presentation to CD8+ T cells, thereby increasing their antitumor efficacy [113]. There are a number of putative biomarkers to pharmacologically control TAM activities including CSF1 and chemokine receptors, costimulatory molecules like CD40, or elements of intracellular pathways like PI3K that all affect the antitumor phenotypes.

Three key characteristics of dendritic cells (DCs) are essential for eliciting a robust and sustained antitumor response: effective migration between lymphoid and non-lymphoid tissues, cross-presentation of tumor-associated antigens (TAAs) to CD8^+^ cytotoxic T lymphocytes to initiate strong effector responses against the tumor, and the release of chemokines and cytokines to regulate the overall immune response and T-cell homing [114]. This makes DCs promising targets for cancer immunotherapy. Higher densities of DCs, particularly classic dendritic cells, within the TME are associated with improved prognosis in ovarian carcinoma, lung cancers, and breast cancers [115,116,117]. However, the TME can disrupt DC functions through various mechanisms. These include reduced production of chemoattractants (e.g., CCL4 and CCL5), which hampers DC recruitment to the tumor site, and decreased levels of survival signals like FMS-like tyrosine kinase 3 ligand (FLT3L), crucial for DC differentiation and viability [118]. These disruptions result in insufficient T-cell activation and may induce T-cell tolerance to TAAs [118].

**Figure 4:**
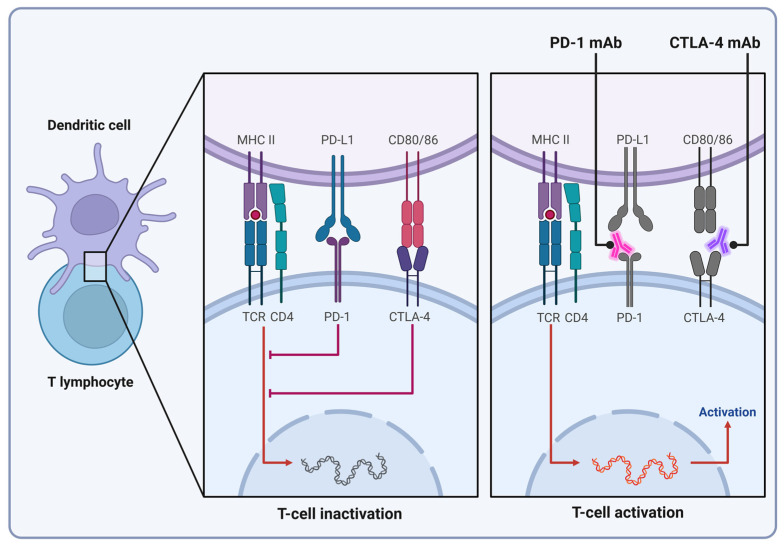
Schematic overview of the prototype immune checkpoint inhibitors. Engaging the immune checkpoint effector PD-1 receptor of T-lymphocytes by the PD-L1 ligand of dendritic cells results in suppression of both the innate and adaptive immunity. Cancer cells can exert this immune paralysis by the expression of PD-L1. This interaction can be blocked by the PD-1 monoclonal antibody (PD-1 mAb). In addition to PD-1/PDL-1, the cytotoxic T-lymphocyte antigen-4 (CTLA-4) ligation by its ligand B7, expressed on antigen-presenting cells, triggers an inhibitory response of T-lymphocyte activity. CTLA-4 targeting monoclonal antibodies (CTLA-4 mAb) can disrupt this interaction. Thus, pharmacological targeting of the PD-1/PD-L1 and CTLA-4/B7 complexes is believed to deliberate T-cell activity from cancer-mediated paralysis and serve as a potential tumor-agnostic targets.

Current cancer immunotherapies targeting T cells involve two main strategies: enhancing the antitumor activity of T cells by inhibiting immune checkpoints and boosting adaptive immunity through the adoptive transfer of genetically engineered T cells, such as those with chimeric antigen receptors (CARs) or modified T-cell receptors [119]. Several negative regulators, or checkpoint molecules, control T-cell activation to prevent immune system overactivity. The prototype of them is the pair of cytotoxic T lymphocytes programmed death receptor 1 (PD-1) and its ligand (PD-L1) (Figure 4). Unlike the canonical tumor-driver biomarkers, neither PD-1 nor PD-L1 are mutated in the context of PD-L1-positive neoplasms. Still, distinct cancer types harboring apparently unrelated tumor-driver mutations have been demonstrated to be positive for PD-L1 expression, making the PD-1/PD-L1 axis an excellent tumor-agnostic target [120,121]. Elevated expression of PD-L1 of cancer cells paralyzes tumor-infiltrating lymphocytes through engaging their PD-1 receptor, resulting in suppression of both the innate and adaptive antitumor immunity. PD-L1 positivity has been documented in a wide range of neoplasms including head and neck cancers, NSCLC, and thyroid, tongue, and pancreatic cancers [120,121]. In many of these neoplasms, elevated PD-L1 expression is thought to be associated with the repression of miR-197, suggesting that the blockade of a transcriptional regulatory mechanism plays a part in the induction of PD-L1-encoding *CD274* in certain transformed cells [121]. *BRAF* mutation-positive colorectal cancers have also been shown to have increased PD-L1 expression, suggesting that the MAPK pathway is involved in the regulation of *CD274* expression in the intestinal epithelium [122,123]. Colorectal cancers, similar to endometrial and gastric tumors, frequently show accumulation of mutations in *MLH1*, *MSH2*, *MSH6* or *PMS2*, genes that encode elements of mismatch repair, typically resulting in a high degree of microsatellite instability [73]. Since these neoplasms have characteristic increases in the number of tumor-infiltrating lymphocytes, their PD-L1 status has fundamental importance in the tumor-agnostic design of their therapeutic approach [124,125,126].

In addition to PD-1/PDL-1, cytotoxic T-lymphocyte antigen-4 (CTLA-4) is another immune checkpoint effector that has recently emerged as a tumor-agnostic biomarker [127]. Physiologically, ligation of CTLA-4 by its ligand B7, expressed on antigen-presenting cells, triggers an inhibitory response of T-lymphocyte activity [127]. Thus, pharmacological targeting of the CTLA-4/B7 complex is believed to deliberate T-cell activity from cancer-mediated paralysis and serve as a potential tumor-agnostic target.

An alternative coreceptor that also participates in T-cell activation is lymphocyte activation gene-3 (LAG-3). LAG-3, a member of the immunoglobulin superfamily, is expressed on the surface of immune cells and actively suppresses T-cell proliferation and effector T-cell function [128]. LAG-3 is structurally and functionally similar to CD4, which ligates with various binding partners, including major histocompatibility complex class II. LAG-3 ligation greatly inhibits CD8^+^ T cells due to their higher LAG-3 expression. Although LAG-3 is often co-expressed with PD-1 on tumor-infiltrating lymphocytes, they are distinct immune checkpoint inhibitors that can be regulated and expressed independently. Consequently, LAG-3 is often upregulated in various neoplasms, including melanoma, to mediate T-cell exhaustion [128,129]. Besides PD-1, CTLA4, and LAG3, other immune checkpoint molecules like TIM3 and TIGIT have also been identified as potential tumor-agnostic targets for T-cell-mediated cancer immunotherapy [130,131,132].

Compared to healthy tissues, tumor vasculature is often irregular and dysfunctional, exhibiting heterogeneous vascular permeability. This results from several morphological and functional changes, including high endothelial cell (EC) proliferation rates, reduced cellular tight junctions, abnormal pericyte coverage, and increased extracellular matrix (ECM) deposition [133]. These vascular abnormalities can lead to inefficient oxygen delivery, creating a hypoxic environment within the tumor, which is associated with increased cancer aggressiveness [134]. Additionally, the dysfunctional vessels can selectively block the infiltration of specific immune cells, such as cytotoxic T lymphocytes, and significantly hinder the delivery and distribution of therapeutic agents [135]. Consequently, targeting tumor vasculature has become a major focus in the study of the TME. Efforts have mainly concentrated on depleting vasculature using antiangiogenic therapies and enhancing drug and immune cell delivery through vessel normalization [136,137,138,139,140,141,142].

The extracellular matrix (ECM) is a complex network of proteins and macromolecules, such as collagens, glycoproteins, elastin, fibronectins, and proteoglycans, which cells secrete into the space between them [143]. ECM composition varies greatly among different organs, and in comparison to healthy tissues, tumor ECM is characterized by increased density, coverage, and stiffness. Within the tumor microenvironment, multiple cell types contribute to ECM production. Beyond providing structural support, the ECM plays a crucial role in regulating the behavior of both cancer cells and other cells within the TME. For example, heightened stiffness of the surrounding tissue can trigger the epithelial-to-mesenchymal transition in cancer cells, a process associated with increased tumor invasiveness, stemness, and metastasis [144]. Moreover, the abnormal accumulation of ECM interferes with therapeutic effectiveness by physically impeding the penetration of drugs and activating signaling pathways like integrin and focal adhesion kinase, which promote cell survival and resistance to chemotherapy [145]. Furthermore, specific patterns of ECM-related gene expression have been linked to poor patient prognosis and resistance to therapy across various cancer types [146,147,148]. In pediatric osteosarcoma, elevated expression of ECM remodeling genes like desmoplakin or SPARCL1 contributes to chemotherapy resistance [146], while in breast cancer, high levels of genes like TWIST are associated with a worse outcome [147,148]. Many research efforts have focused on strategies to degrade ECM components, such as using collagenases or hyaluronidases to improve drug distribution [149]. Alternatively, given the dynamic nature of ECM remodeling by enzymes produced within the TME, an unconventional therapeutic approach involves directly targeting the synthesis of ECM components. This could be achieved by inhibiting key signaling pathways like TGFβ or HIF1α, which promote ECM production, or by targeting the modifying enzymes necessary for ECM component synthesis, secretion, and maturation [150,151].

Cancer-associated fibroblasts (CAFs) are pivotal in shaping the tumor microenvironment by producing ECM molecules. These fibroblasts facilitate tumor growth through several mechanisms. Not only do they deposit ECM, but they also produce matrix remodeling enzymes that promote tumor invasion, metastasis, and resistance to therapies. Furthermore, CAFs secrete various cytokines, exosomes, and growth factors, such as leukemia inhibitory factor (LIF) and growth differentiation factor 15 (GDF15), which further drive tumor growth and invasion [152,153,154]. The influence of CAFs extends beyond tumor cells to other TME components, including the vasculature and immune cells. For instance, CAF-derived VEGF stimulates angiogenesis, while cytokines like IL6, CXCL9, and TGFβ modulate T-cell responses [155,156]. This complex interaction network highlights the crucial role of CAFs in cancer progression and the dynamic nature of the TME.

Developments in our understanding of cellular transformation and the revolution in molecular biology-based diagnostic technologies led to the current paradigm shift in clinical oncology from the former histopathological classification of human neoplasms to the molecular aberration-focused approach we now know as the tumor-agnostic philosophy.

## 3. Tumor-Agnostic Treatment Philosophy in Current Clinical Oncology

The identification of key tumor-driver mutations led to the concept of novel anticancer strategies on the basis of targeting common biomarkers of human neoplasms. Regulatory approvals underpinned this paradigm shift, with the FDA greenlighting new therapies tailored for tissue-agnostic indications [7,9].

### 3.1. Tumor-Agnostic Applications in Contemporary Clinical Practice

#### Immunotherapies

Pembrolizumab, the first FDA-approved tumor-agnostic medicine, is a humanized monoclonal antibody that directly binds to PD-1 of T lymphocytes, interfering with the ligation of PD-L1 and preventing the tumor cell-mediated inhibition of PD-L1^+^ lymphocytes [126,157]. In clinical trials, its use showed impressive results, with an objective response rate (ORR) of 40%, a disease control rate (DCR) of 90%, and progression-free survival of 78% in mismatch repair-deficient colorectal cancer patients [158]. In endometrial, small bowel, and gastric cancers, as well as in cholangiocarcinoma patients, the same measures showed 67%, 71%, and 71% for progression-free survival (PFS), ORR, and DCR, respectively [157,158]. Thus, it is not surprising that it was granted accelerated approval by the United States Food and Drug Administration (FDA) for patients with unresectable or metastatic microsatellite instability-high (MSI-H) or mismatch repair-deficient (dMMR) positive tumors in 2017 [159]. The approval was based on combined data from five single-arm trials, involving a total of 149 patients diagnosed with MSI-H/dMMR cancers, with the majority (90 patients) having colorectal cancer. Pembrolizumab exhibited manageable adverse reactions, and recent findings from the phase II KEYNOTE-158 study confirmed its robust and durable antitumor efficacy. A complete response was observed in a wide range of tumor types, including small intestine, gastric, ovarian, endometrial, and cholangiocarcinomas. The response was durable, with a 47.5-month median duration of the clinical response [126].

Following the approval in 2017, continuous evaluation of pembrolizumab has also revealed its efficiency in treating solid tumors with high metastatic tumor mutational burden (TMB-H) [160]. High TMB is defined as the occurrence of more than 10 somatic mutations per megabase and is seen in various histologically unrelated human neoplasms [160,161]. Based on the rationale that these tumors might be treated more efficiently using immune checkpoint inhibitors than targeting their complex and likely dynamic mutational pattern, pembrolizumab was granted a second accelerated approval by the FDA for patients with TMB-H-positive, unresectable, or metastatic solid tumors in 2020 [160,161].

The rapid and successful implementation of pembrolizumab in practical oncology has led to the development of additional monoclonal antibodies targeting biomarkers involved in the evasion of antitumor immune responses (Table 3). One of these candidates is PD-L1 itself, and humanized monoclonal anti-PD-L1 antibodies, e.g., atezolizumab, have already been developed to block PD-L1’s engagement with inhibitory T-cell proliferation regulators PD-1 and B7, thus preventing T-cell depletion and increasing T-cell-mediated immunity against neoplasms [162,163]. Atezolizumab has already received FDA approval as the first-line treatment for patients with metastatic non-small-cell lung cancer if they express high levels of PD-L1 or have PD-L1-stained tumor-infiltrating immune cells covering more than 10% of the tumor area and do not possess genomic mutations or rearrangements in epidermal growth factor receptor (*EGFR*) or *ALK*. Drug efficacy was evaluated in the IMpower110 clinical trial that compared platinum-based chemotherapy to atezolizumab and documented an impressive improvement in overall survival (OS), with a median OS of 20.2 months compared to the 12.1-month OS of patients receiving chemotherapy only [164].

In addition to PD-1/PDL-1, antibody-mediated targeting of cytotoxic T-lymphocyte antigen-4 (CTLA-4) has also been developed [127]. Targeted by the human monoclonal antibody ipilimumab, the antibody-mediated ligation of CTLA-4 blocks its interaction with its ligand B7, expressed on antigen-presenting cells, and the blockade of this interaction is believed to facilitate cytotoxic T-lymphocyte activity [127,165]. On that basis, in 2020, the FDA approved ipilimumab as the first-line treatment for patients with non-small-cell lung cancers in combination with nivolumab (another PD-1 inhibitor) if immunohistochemistry confirms PD-L1 expression ≥1% and tumor panel investigations show no presence of *EGFR* or *ALK* mutations [166]. Drug efficacy was observed through the CHECKMATE-227 trial, with an overall response rate of 36%. The trial also showed 23% of the patients surviving at least 5 years, which is remarkably significant considering the poor prognosis associated with non-small-cell lung cancers, where the 5-year survival rate is merely 7% [166,167].

Since CTLA-4 and PD-1 inhibitors act on immune checkpoint regulators in a complementary manner, it is not surprising that their use in combination frequently provokes immune-related adverse reactions. Although the majority of these side effects are mild to moderate and can be well managed with supportive treatments without discontinuing nivolumab and ipilimumab, there have been reports of fatalities due to autoimmune myocarditis, colitis, and myasthenia gravis associated with the use of them as well [168]. The clinical management of these severe side effects is particularly challenging due to the observation that their onset can occur even after drug clearance [168]. To address these challenges, a recent modeling study, based on multiomics investigations, found a close correlation between the number of InDel and point mutations of cancer cells, the amount of tumor-infiltrating lymphocytes, and the risk of immune-related adverse events, highlighting the importance of mutational evaluations of neoplasms before indicating combinatorial immune checkpoint therapies. This modeling also suggests that upon the elevated predicted risk of immune-related adverse events, a reduction in the dosage of immune checkpoint inhibitors circumvents the unfolding of their severe side effects [169].

The use of bispecific recombinant monoclonal antibodies, like tebotelimab, might be a solution to reduce the severe side effects of combinatorial immune checkpoint inhibitor therapies. Tebotelimab targets both PD-1 and LAG-3 either simultaneously or independently. Its bivalent design ensures individual blocking of PD-1 and LAG-3 from their respective ligands, irrespective of their co-expression. Binding to either PD-1 or LAG-3 promotes cell adherence, thereby increasing the local antibody concentration. Since tumor cells often compensate for the initial pharmacological blockade by inducing secondary checkpoint mediators, the bispecific nature of tebotelimab allows for the effective inhibition of the secondary checkpoint mediator on the same cell [128]. Tebotelimab induces greater T-cell activity and IFN-γ production than monophasic PD-1 or LAG-3 therapies alone or in combination, while exhibiting better tolerance. The potential benefit of dual inhibition of PD-1 and LAG-3 treatments in patients resistant to PD-1 inhibitors, however, is yet to be investigated [128].

Another plausible way to address the challenges that the use of immune checkpoint inhibitors might pose is the use of these medicines in combination with other agents that exploit distinct mechanisms of action (Table 4). Indeed, besides the rapid development of immune checkpoint-targeting medicines, direct tumor drivers have also served as subjects for targeted drug developments. This class of tumor-agnostic agents typically includes small-molecule pharmacological inhibitors that show a high degree of specificity to their dedicated tumor driver targets, so they can complement immune checkpoint-targeting monoclonal antibodies in combinatorial treatments. Indeed, a recent randomized phase III trial investigating the efficacy of the combination of PD-1, CTLA-4, and BRAF/MEK inhibitors dabrafenib and trametinib in melanoma patients showed significant clinical benefits [170].

Dabrafenib and trametinib have been developed to selectively inhibit mutated BRAF and mitogen-activated protein kinase 1 and 2 (*MEK1* and *MEK2*) through competitive ATP-binding site inhibition and allosteric blockade, respectively. *BRAF* mutations have high prevalence in melanomas, papillary thyroid cancers, and non-small-cell lung cancers, although it was also detected in gliomas and gastric cancers. Interestingly, BRAF inhibitors alone, paradoxically, activate the MAPK pathway, fueling the fear of secondary neoplasms. In combination, however, dabrafenib and trametinib effectively block the MAPK pathway and suppress the proliferation of BRAF V600E cells [171,172,173]. Accordingly, dabrafenib and trametinib has been approved for use in combination for patients with anaplastic thyroid cancer (ATC) harboring the V600E BRAF mutation, when no other satisfactory treatments are available [174].

Competitive ATP-binding inhibitors selective for mutated kinases have now been developed for various cytoplasmic and receptor kinases and have already found their way into everyday clinical practice. Larotrectinib, for instance, a highly selective NTRK inhibitor, has already been approved for patients with solid tumors harboring chimeric *NTRK* genes without a known acquired resistance mutation [175,176]. Clinical data suggest that Larotrectinib can pass the blood–brain barrier, an important aspect considering the prevalence of NTRK mutations among neoplasms [177]. Indeed, chimeric mutant *NTRK*s were found in a variety of adult and pediatric neoplasms including infantile fibrosarcoma, pediatric papillary thyroid carcinoma, and NSCLC, of which the latter ones can form metastases within the CNS [175,177]. Unfortunately, Larotrectinib is believed to have high affinity to P-Glycoprotein (P-GP), a well-characterized ABC transporter that is key to the efflux of the blood–brain barrier, suggesting a lower maximum concentration of the agent achievable within the CNS [178]. Other competitive ATP-binding NTRK inhibitors, like entrectinib, however, have weaker P-GP affinities, allowing higher brain-to-plasma concentration ratios [178]. Indeed, clinical data showed a complete response in patients with brain metastases of NTRK-positive lung cancer as well as significant disease reduction in NTRK-positive glioneuronal tumors following entrectinib exposure, highlighting its effectiveness against both primary and metastatic brain neoplasms [179]. Entrectinib, in addition, has inhibitory effects on both ALK and the receptor tyrosine kinase ROS proto-oncogene 1 (ROS1), extending its therapeutic applications beyond NTRK-mutated tumors [62,180]. Indeed, in clinical trials, efficacy has been documented in both NTRK-mutated and mutated ROS1-harboring neoplasms [62].

Recently, another highly selective ATP-competitive inhibitor for RET, selpercatinib, has been approved by the FDA for treating tumors positive for mutant *RET* alleles [181]. Positive responses to selpercatinib have been reported in various tumors including pancreatic adenocarcinoma, salivary, colorectal, ovarian, breast, and small intestine cancers, soft-tissue sarcomas, bronchial carcinoids, and cholangiocarcinomas alike [181]. Further details on the FDA-approved tumor-agnostic drugs are provided in Table 3.

**Table 3:** FDA-approved tumor-agnostic medicines currently implemented in clinical practice. PD-1, programmed cell death protein 1; PD-L1, programmed death ligand 1; CTLA-4, Cytotoxic T-lymphocyte associated protein 4; NTRK, neurotrophic receptor tyrosine kinase; RET, Rearranged during Transfection; ORR, overall response rate; DOR, duration of response; 1-y PFS, 1-year progression-free survival; mDOR, median duration of the response.

Drug	Target	Edvidence for Approval	Common Adverse Effects	FDA Approval	Reference
Pembrolizumab	PD-1	Combined data consisting of 149 patients enrolled across five single-arm studies. ORR: 39.6% (31.7–47.9%; 95% CI); mDOR ≥ 6 months in 78% of patients	Reported in ≥20% of patients including cough, fatigue, nausea, rash, pruritus, decrease appetite, arthralgia, diarrhea, and constipation	2017	[159,182]
Dostarlimab	PD-1	Analysis derived from an ongoing, open label, single-arm multi-cohort phase I study. ORR: 43.5% (34.0–53.4%; 95% CI); mDOR: was not reached.	Reported in ≥20% of patients including nausea, diarrhea, fatigue, anemia, and constipation	2021	[183,184]
Larotrectinib	NTRK	Combined data consisting of 55 patients enrolled across three single-arm studies. ORR: 75% (61–85%; 95% CI); 1-y PFS: 55%; mDOR: was not reached.	Reported in ≥20% of patients including nausea, dizziness, vomiting, increased AST, diarrhea, fatigue, increased ALT, cough, and constipation	2018	[176,185]
Entrectinib	NTRK	Combined data consisting of 54 patients enrolled across three single-arm studies. ORR: 57% (43.2–70.8%; 95% CI); mDOR: 10 months (7.1-not estimable; 95% CI).	Reported in ≥20% of patients including nausea, dizziness, vomiting, diarrhea, fatigue, edema, dysesthesia, dyspnea, myalgia, cognitive impairment, increase weight, vision disorders, arthralgia, vomiting, pyrexia, cough, and constipation	2019	[186,187]
Dabrafenib plus Trametinib	BRAFV600E	Combined data consisting of 167 patients (131 adults, 36 pediatric patients) enrolled across three single-arm studies. ORR adults: 41% (33–50%; 95% CI); ORR children: 25% (12–42%; 95% CI); DOR: ≥6 months for 78% of patients, ≥24 months for 44% of patients.	rash, headache, hemorrhage, cough, myalgia, nausea, constipation, vomiting, diarrhea, pyrexia, fatigue, chills, peripheral edema, and arthralgia	2022	[173,174,188,189,190]
Selpercatinib	RET	Analysis derived from a multi-cohort single-arm phase I/II study. ORR: 44% (28–60%; 95% CI); mDOR: 24.5 months (9.2-not evaluable; 95% CI).	Reported in ≥20% of patients including dry mouth, edema, fatigue, hypertension, diarrhea, abdominal pain, constipation, rash, nausea, and headache	2022	[181]
Atezolizumab	PD-L1	Analysis derived from a multicenter, randomized, open-label trial. ORR: 38% (29–48%; 95% CI); mDOR: 20.2 months (16.5-not evaluable; 95% CI).	Reported in ≥20% of patients including fatigue, decrease appetite, nausea	2020	[164,191]
Nivolumab plus Ipilimumab	PD-1 and CTLA-4	Analysis derived from a randomized, open label, muti-part trial. ORR: 36% (31–41%; 95% CI); mDOR: 23.2 months (15.2-32.2; 95% CI).	Reported in ≥20% of patients including decreased appetite, musculoskeletal pain, diarrhea/colitis, dyspnea, fatigue, rash, cough, pruritis, nausea, and hepatitis	2020	[166,192]
Fam-trastuzumab deruxtecan-nxki	HER2	Individual analyses for each trial consisting of 192 patients enrolled in one of three multicenter trials: DESTINY-PanTumor02, ORR: 51.4% (41.7–61%; 95% CI); mDOR: 19.4 months (range 1.3, 27.9+). DESTINY-Lung01, ORR: 52.9% (27.8–77%; 95% CI); mDOR: 6.9 months (range 4.0, 11.7+). DESTINY-CRC02, ORR 46.9% (34.3–59.8%; 95% CI); mDOR: 5.5 months (range 1.3+, 9.7+)	Reported in ≥20% of patients including vomiting, alopecia, constipation, decreased appetite, nausea, fatigue, leukopenia, cough, anemia, diarrhea, and thrombocytopenia	2024	[193,194]
Trastuzumab	HER2	Analysis derived from a phase 3 clinical trial. ORR: 50% (*p* < 0.001); mDOR: 9.1 months (*p* < 0.001)	Most important adverse event reported was cardiac dysfunction	1998	[195]
Gefitinib	EGFR	Analysis derived from a randomized, double-blind, phase II, multicenter trial. ORR: 10.6% (6.0–16.8%; 95% CI); mDOR: 7.0 months (range 4.6–18.6+)	Reported in ≥5% of patients including diarrhea, rash, and acne	2003	[196]
Imatinib	BCR/ABL	Analysis derived from phase I and phase II clinical studies. OS: 90.8% (88.3‖93.2%; 95% CI)	Most reported adverse event includes edema, rash, gastrointestinal disturbances, and musculoskeletal complaints	2001	[197,198]

**Table 4:** FDA-approved combination tumor-agnostic therapies. HRR, homologous recombination repair; PD-L1, Programmed death ligand 1; HER2, human epidermal growth factor receptor 2; TNBC, triple-negative breast cancer.

Combination Therapies	Indications	Reference
Nivolumab with Ipilimumab	Hepatocellular carcinoma, malignant pleural mesothelioma, melanoma	[199,200,201]
Pembrolizumab with lenvatinib	Endometrial carcinoma, renal cell carcinoma	[202,203]
Daratumumab and hyaluronidase-fihj with pomalidomide and dexamethasone	Multiple myeloma	[204]
Isatuximab-irfc, carfilzomib and dexamethasone	Multiple myeloma	[205]
Nivolumab with cabozantinib	Renal cell carcinoma	[206]
Pembrolizumab with axitinib	Renal cell carcinoma	[207]
Avelumab with axitinib	Renal cell carcinoma	[208]
Avelumab with chemotherapy	Urothelial carcinoma	[209]
Atezolizumab with bevacizumab	Hepatocellular carcinoma	[210]
Atezolizumab with chemotherapy and bevacizumab	Non-squamous, non-small-cell lung cancer	[211]
Atezolizumab, cobimetinib and vemurafenib	BRAF V600 unresectable or metastatic melanoma	[212]
Enfortumab vedotin-ejfv with pembrolizumab	Urothelial cancer	[213]
Tremelimumab with durvalumab	Hepatocellular carcinoma	[214]
Carfilzomib and daratumumab with dexamethasone	Multiple myeloma	[215]
Lenalidomide with tafasitamab-cxix	Diffuse large B-cell lymphoma	[216]
Lenalidomide with rituximab	Follicular and marginal zone lymphoma	[217]
Pertuzumab, trastuzumab, and hyaluronidase-zzxf	HER2-positive breast cancer	[218]
Ramucirumab with erlotinib	Metastatic non-small-cell lung cancer	[219]
Olaparib with bevacizumab	Ovarian, fallopian tube, or primary peritoneal cancers	[220]
Ibrutinib with rituximab	Chronic lymphocytic leukemia	[221]
Encorafenib with cetuximab	Metastatic colorectal cancer with a BRAF V600E mutation	[222]
Relatimab with Nivolumab	Melanoma	[223]
Tucatinib with trastuzumab	Colorectal cancer	[224]
Durvalumab with chemotherapy	Endometrial cancer, biliary tract cancer, extensive-stage small-cell lung cancer	[225,226,227]
Ponatinib with chemotherapy	Philadelphia chromosome-positive acute lymphoblastic leukemia	[228]
Zanubrutinib with obinutuzumab	Relapsed or refractory follicular lymphoma	[229]
Nivolumab with cisplatin and gemcitabine	Urothelial carcinoma	[230]
Osimertinib with platinum-based chemotherapy	Epidermal growth factor receptor-mutated non-small-cell lung cancer	[231]
Pembrolizumab with chemoradiotherapy	FIGO 2014 Stage III-IVA cervical cancer	[232]
Pembrolizumab with trastuzumab, fluoropyrimidine, and platinum-containing chemotherapy	HER2-positive gastric or gastroesophageal junction adenocarcinoma	[233]
Pembrolizumab with chemotherapy	HER2-negative gastric or gastroesophageal junction adenocarcinoma, biliary tract cancer, cervical cancer, high-risk early-stage TNBC, esophageal carcinoma, head and neck squamous cell carcinoma	[234,235,236,237,238,239]
Encorafenib with binimetinib	Non-small-cell lung cancer with a BRAF V600E mutation	[240]
Trifluridine and tipiracil with bevacizumab	Colorectal cancer	[241]
Dostarlimab-gxly with chemotherapy	Endometrial cancer	[242]
Quizartinib with chemotherapy	Acute myeloid leukemia	[243]
Tremelimumab with durvalumab and platinum-based chemotherapy	Non-small-cell lung cancer	[244]
Brentuximab vedotin with chemotherapy	Pediatric patients with classical Hodgkin lymphoma	[245]
Cemiplimab-rwlc with platinum-based chemotherapy	Non-small-cell lung cancer	[246]
Dabrafenib with trametinib	Solid tumors with BRAF V600E mutation	[174]
Nivolumab with chemotherapy or with ipilimumab	Esophageal squamous cell carcinoma	[247]
Rituximab with chemotherapy	Pediatric patients (≥6 months to <18 years) with previously untreated, advanced stage, CD20-positive diffuse large B-cell lymphoma, Burkitt lymphoma, Burkitt-like lymphoma, or mature B-cell acute leukemia	[248]
Nivolumab with chemotherapy	Gastric cancer, esophageal adenocarcinoma	[249]
Margetuximab-cmkb with chemotherapy	HER2-positive breast cancer	[250]
Naxitamab with granulocyte-macrophage colony-stimulating factor	High-risk neuroblastoma in bone or bone marrow	[251]
Nivolumab with ipilimumab and chemotherapy	Non-small-cell lung cancer	[252]
Tucatinib with trastuzumab and capecitabine	HER2-positive breast cancer	[253]
Neratinib with capecitabine	HER2-positive breast cancer	[254]

### 3.2. Future Perspectives

Besides the tyrosine kinase and immune checkpoint receptors, significant efforts have been made in developing agents targeting receptors belonging to other functional receptor classes. CD137 (also known as 4-1BB or tumor necrosis factor receptor superfamily member 9), for instance, is a costimulatory molecule transiently expressed on the surface of various leukocytes, including activated T cells and natural killer (NK) cells. Activation of CD137 depends on its ligation with CD137L, a trimer expressed on antigen-presenting cells. This interaction induces downstream pathways that are crucial for antitumor responses [255]. Upon CD137 activation, CD8^+^ cytotoxic T cells receive potent signals promoting their proliferation, cytolytic effector functions, and survival, while CD137 ligation in NK cells fosters cytokine release and cytolytic functions [256,257]. Clinically, CD137 ligation seems to be particularly promising since data suggest that CD137L binding can restore the antitumor capacity of exhausted CD137^+^ CD8^+^ T cells, making CD137 a potential target for tumor-agnostic immunotherapy [258]. Unlike the first developed anti-CD137 IgG4 monoclonal antibody urelumab and the IgG2 monoclonal antibody utomilumab, which have severe hepatotoxicity and weaker agonistic functions, respectively, the novel human recombinant IgG4 monoclonal antibody, HuB6, demonstrated effective antitumor activity without systemic toxicity in distinct animal tumor models [255,258]. HuB6 has similar affinity for human 4-1BB to that of utomilumab but binds to a unique epitope within CD137 and shows greater agonistic activity. Despite being IgG4 recombinants, like urelumab, HuB6 exhibits higher safety profiles in vivo, which can be attributed to variations in epitope binding. Urelumab attaches to an epitope that orients its Fc domain for easy ligation with FcγR, potentially enhancing antibody-dependent cell-mediated cytotoxicity and complement-dependent cytotoxicity [258]. However, stronger immune cell activation, especially in non-tumor tissues, can trigger autoimmune responses leading to severe hepatotoxicity. In contrast, utomilumab binds to an epitope closer to the cell surface, positioning the antibody parallel to the membrane, thereby hindering its interaction with FcγR, potentially leading to its limited agonistic function. HuB6, on the other hand, presents with moderate interaction with FcγR, ranging between urelumab and utomilumab [258]. This moderate binding provides sufficient affinity to induce downstream signaling and immune activation without overstimulating the immune system. Furthermore, IgG4 generally elicits a greater interaction with immune cells via FcγR interaction than IgG2, which may enhance the agonistic activity of HuB6 despite its similar affinity for human 4-1BB to utomilumab [258]. In addition to being an efficacious monotherapy, HuB6 also exerted an enhanced antitumor effect in combination with other immunotherapies, such as anti-PD-1, indicating broader clinical applications. This promising antitumor efficacy has led to HuB6’s approval for clinical trials investigating various malignant types in addition to colorectal cancer [258].

Understanding the correlation between the targeted epitopes and the clinical efficacy of monoclonal antibodies, like in the case of targeting CD137, is critical to improve the clinical outcome of these novel tumor-agnostic methodologies. Human epidermal growth factor receptor 2 (HER2), for instance, has been heavily studied as a target for monoclonal antibodies, since its overexpression is not only found in breast and gastric neoplasms, but it might be present in non-small-cell lung cancer and colorectal cancer as well [259]. In addition, *HER2* mutations have been associated with a more aggressive tumor progression in younger populations [193]. As part of these efforts, a bispecific monoclonal antibody, Zenocutuzumab, has also been recently developed for treating cancers with mutated *HER2*. Zenocutuzumab is a humanized immunoglobin that contains two distinct F_ab_ arms targeting both HER2 and HER3, employing the so-called dock and block mechanism. The HER2-targeting arm initially binds to HER2 which, usually, is expressed more abundantly on the cell surface. This not only increases the local concentration of the antibody but also positions the HER3-targeting arm to block neuregulin 1 (NGRI) from binding to HER3. NRG1 primarily binds to HER2 and HER3, leading to heterodimer formations and subsequent stimulation of oncogenic signaling. Mutated *NGR1* has been associated with numerous malignancies including lung, breast, ovarian, prostate, and pancreas [260]. Thus, the Zenocutuzumab-mediated blockade of NGR1 ligation prevents the conformational change of HER3 required for heterotrimerization with HER2 and NGR1, consequently inhibiting downstream tumor-inducing signaling. So far, Zenocutuzumab has been tested in three patients suffering from *NRG1* fusion-positive cancers. Two of the patients with pancreatic cancer experienced tumor regression and continued the therapy for 19 and 11 months, respectively. The third patient with driver-negative non-small-cell lung cancer experienced a partial response [260]. The drug is overall well tolerated among the patients, with diarrhea being the most reported adverse drug reaction. As of April 2024, a global, multicenter phase I/II clinical trial is pending to evaluate the efficacy of Zenocutuzumab further.

Parallel HER2-directed antibodies are also being developed to serve as delivery vehicles for various anticancer compounds. Indeed, as of 2024, the FDA granted accelerated approval to fam-trastuzumab deruxtecan-nxki for patients with unresectable or metastatic *HER2*-positive tumors, even if already pretreated or with no alternative treatment options available [194]. Trastuzumab deruxtecan is an antibody–drug conjugate consisting of a humanized anti-HER2 antibody linked with a potent cytotoxic drug via a cleavable, peptide-based linker. Once the drug binds to HER2-expressing tumor cells, the linker is cleaved, releasing the cytotoxic drug. The peptide-based linker is thought to be selectively cleaved by lysosomal enzymes, which are upregulated in the tumor microenvironment. This drug inhibits topoisomerase I through the stabilization of DNA–topoisomerase complexes, resulting in double-strand breaks and subsequent destruction of neoplasms. It is believed that the localized effect of the drug limits systemic toxicity in normal cells [259,261].

With our expanding knowledge of the molecular constellation of human neoplasms, new concepts continuously emerge for the development of novel therapeutic modalities. In addition to directly leveraging our immune surveillance against neoplasms, emerging therapeutic treatments focus on interfering with additional aspects of the cellular physiology, like apoptosis. One of these candidate targets is myeloid cell leukemia-a (MCL1), a member of the BCL2 family that regulates intrinsic apoptotic pathways via a series of signaling cascades or through mitochondrial apoptogenic factors [262]. Given the pivotal role of the BCL2 family, it is not surprising that its disruption would contribute to tumorigenesis and drug resistance. Indeed, MCL1 is often upregulated in various solid tumors, particularly in melanomas where its increased expression correlates with poor response to cancer therapies due to upregulation of Myeloid-derived suppressor cells (MDSCs) [262]. An increased number of MDSCs are believed to contribute to the immune evasion of tumor cells by altering the tumor microenvironment through the secretion of immunosuppressive agents such as interleukin-10, VEGF, and reactive oxygen species and increased expression of cell surface receptors that suppress T-cell activity [262]. On this basis, recent investigations into BH3 mimetics, a new class of drug consisting of small-molecule inhibitors that selectively inhibits anti-apoptotic members through mimicking the action of pro-apoptotic family members, have shed light on potential therapeutic use of targeting apoptosis regulators like MCL1. Indeed, S64315, an MCL1 inhibitor, has gained attention for its hypothesized ability to decrease MDSC frequency through impeding MCL1 expression. Studies have shown that S64315 monotherapy reduces MDSC frequency in mouse models, leading to improved CD8^+^ T-cell function. Moreover, S64315 demonstrated greater efficacy in combination therapy with anti-PD-1, effectively inhibiting melanoma growth and enhancing T-cell activity. However, further research is needed to delineate optimal treatment strategies using MCL1 inhibitors, since their current generation shows dose-dependent cardiac toxicity due to high expression levels of MCL1 in cardiomyocytes [262].

Focusing on the TME, many macrophage-targeted therapies are currently in clinical trials for various tumor types. Inhibition of the CSF1 receptor is believed to deplete TAMs and/or modify their functions within the TME, while a pharmacological blockade of CC-motif chemokine ligand 2 (CCL2) or CC-chemokine receptor 2 (CCR2) might prevent TAM recruitment into the TME [263,264]. CD47/SIRPα complex antagonists are also in the test phase to enhance the TAM-mediated phagocytosis of cancer cells as well as inhibitors of PI3Kγ and TREM2 proteins to reprogram TAMs toward antitumor phenotypes [265,266].

Pharmacological intervention of TAM physiology is predicted to affect TME on an intercellular level as well. Indeed, administration of costimulatory molecules, like CD40, is expected to enhance reactivity of the immune system via T-cell activation [267]. To further support T-cell reactivity against tumor cells, strategies to manipulate DCs are also in the development pipeline, including the use of FLT3L to promote DC expansion and survival in vivo or the modulation of DC activity with GM-CSF [268,269]. To expand the DC population, the use of DC vaccines to enhance antitumor immunity is also under investigation [270].

Targeting cancer-associated fibroblasts as part of a strategy for tumor-agnostic anticancer therapy is also the focus of current studies. One promising approach involves inhibiting fibroblast activation protein (FAP), given that FAP-expressing CAFs are linked to immunosuppression in various preclinical models and human samples [156,271,272]. Additionally, since CAF activation and function are regulated by signaling pathways such as Hedgehog, NFκB, CXCR4, FGFR, and TGFβ, specific inhibitors targeting these pathways are currently being evaluated in clinical trials [273]. Alternative strategies to depleting CAFs include reprogramming or normalizing them using vitamin D or vitamin A. For example, treating pancreatic cancer preclinical models with a vitamin D analog has been shown to revert CAFs to their quiescent stellate cell state, thereby enhancing antitumor efficacy [274]. Moreover, recent studies have highlighted that targeting a specific subset of CAFs expressing CD10 and GPR77 can increase chemotherapy sensitivity in breast cancer models [275]. These diverse approaches underscore the potential of CAF-targeted therapies in improving cancer treatment outcomes.

## 4. Discussion

In recent years, there has been a striking rejuvenation in cancer treatment, characterized by a profound shift in therapeutic strategies from targeting specific tumors or molecular traits to concentrating on specific molecules irrespective of tumor origin. Throughout this transformative period, the trajectory of cancer treatment was intimately intertwined with the relentless progress of diagnostic technologies, from the inception of microscopy in pathology to the cutting-edge tools of modern molecular biology [276,277].

The journey into the realm of precision oncology began in the late 1980s when the discovery of human epidermal growth factor-2 (HER2) overexpression or amplification in breast cancer emerged as a pivotal breakthrough. Initially identified through immunohistochemistry (IHC) and subsequently confirmed by in situ hybridization (ISH) techniques, this revelation paved the way for the development of trastuzumab, a humanized antibody targeting HER2. Its groundbreaking approval by the Food and Drug Administration (FDA) in 1998 marked a significant milestone in cancer treatment [278,279]. Following this achievement, strides were made in the development of targeted therapies. Noteworthy examples include imatinib, which inhibits the BCR/ABL tyrosine kinase in chronic myeloid leukemia (CML), and gefitinib, targeting the epidermal growth factor receptor (EGFR) in non-small-cell lung cancer (NSCLC).

A pivotal advancement in reshaping cancer treatment paradigms came with the landmark achievement of the first human genome sequencing in 2001. Yet it was the subsequent emergence of NGS techniques around the same period that truly revolutionized the field, enabling rapid, cost-effective, and extensive parallel sequencing of DNA and RNA [279,280]. The widespread integration of modern molecular biology methodologies has provided deeper insights into the intricate molecular landscape of cancer, uncovering a myriad of mutations, some driving selective growth advantages while others remain bystanders. This vast pool of molecular data has undeniably enriched the field of oncology. However, it has also introduced complexities, as distinguishing between driver and non-driver mutations is not always straightforward, and not all driver mutations are amenable to targeted therapies. Nevertheless, in certain cancer types, the identification of molecular subgroups based on specific driver mutations has led to the development of tailored targeted therapies, resulting in significant prognostic improvements [280,281,282,283,284].

The advent of immunotherapy has introduced a formidable new weapon against cancer as well. In select cases, immunotherapy has demonstrated remarkable and enduring responses [285,286], highlighting its potential to engage the immune system and evoke robust tumor responses, including through interactions involving amino acid residues [287].

The expansive growth of precision oncology and immune oncology has ushered in two distinct currents in cancer research: combination therapies and molecular-specific/tumor-agnostic treatments. Recognizing that most cancers arise from a complex interplay of molecular irregularities, investigators have delved into combination therapies, such as pairing chemotherapy with targeted agents, chemotherapy with immunotherapy, or various combinations of immunotherapy agents. The objective is to heighten treatment efficacy and circumvent potential resistance mechanisms, yielding promising outcomes across diverse cancer types [288,289,290,291]. However, while combination therapies have demonstrated notable advantages, their success may not solely stem from synergistic drug interactions. Instead, it could be attributed to a broader treatment spectrum, encompassing different patient subgroups that are responsive to various therapeutic approaches. This approach, however, may entail a “loss of precision”, potentially leading to overtreatment among certain patient subsets [280,292].

On the contrary, molecular-specific/tumor-agnostic therapies have emerged primarily in response to two specific clinical dilemmas. Firstly, they address cases where a tumor exhibits a molecular abnormality for which a targeted therapy exists but is typically used for other tumor types. Secondly, they cater to instances where rare mutations/abnormalities with potential treatment options are identified across various tumor types, including those considered rare or ultra-rare. However, both scenarios often involve small sample sizes, making the traditional approach of single-histology trials impractical. Consequently, there has been a rise in off-label use of molecularly targeted agents, resulting in numerous promising case reports. Yet these reports are inherently biased, as negative outcomes from individual cases tend to be underreported. To address these challenges, master protocols have been devised, particularly histology-agnostic/aberration-specific basket and “n-of-1” trials encompassing multiple subgroups or studies involving patients with similar or diverse diseases to assess the efficacy of one or more therapies [293,294,295,296,297,298]. These protocols have gained considerable traction and have been embraced by regulatory authorities in recent years. They offer a pathway to overcome the limitations posed by small sample sizes, facilitating more rigorous evaluation of novel therapies [279,280,281,294,299,300].

Basket trials epitomize a master protocol approach, scrutinizing the effectiveness of a solitary drug across patients afflicted with diverse diseases but united by a shared molecular anomaly, operating through parallel sub-studies. Rooted in the traditional phase I dose escalation design, these trials pioneer inclusive enrollment, disregarding tissue type, to establish a recommended dosage—a strategy historically employed to pave the way for subsequent phase studies targeting specific histologies. Embracing the notion that biomarkers can prognosticate the response to targeted therapy regardless of tumor histology is pivotal for the advancement of tissue-agnostic treatments [301]. Basket trials not only enable the examination of drugs within molecularly defined subgroups but also validate the predictive prowess of biomarkers. Furthermore, they unravel contextual nuances, recognizing histology as a crucial variable: the disease-specific milieu surrounding a targetable mutation may influence drug efficacy [302]. Variations in tumor types can redefine the significance of molecular anomalies owing to diverse oncogenic pathways and resistance mechanisms. Moreover, histology-specific factors, such as tumor microenvironments, intricately shape drug delivery and immunosurveillance [303].

Despite their significance, basket trials encounter notable challenges. Their rarity often translates into small sample sizes, undermining the robustness of conclusions drawn. Subgroups may suffer from scant participant numbers, further complicating result reliability [281]. Predominantly conducted as phase I/II trials, they primarily focus on activity and safety, sidelining efficacy assessments. While randomized controlled trials remain indispensable for drug approval, in the case of exceedingly rare conditions or drugs demonstrating promising early efficacy, a randomized approach may prove impractical or dispensable for registration [303,304,305]. Basket trials offer substantial value in exploring rare cancers or mutations characterized by a single, low-complexity driver mutation. Conversely, common cancers exhibit a multifaceted molecular landscape, governed by numerous genomic, transcriptomic, and proteomic alterations, intricately shaping therapeutic responses [281].

The “n-of-1” trial design, a recent addition to oncology research, aims to delve into personalized, molecular-driven treatment strategies tailored to each patient [280]. In this innovative approach, patients are administered a bespoke combination therapy that aligns with their unique molecular profile, necessitating the involvement of an expert tumor molecular board to accurately interpret individual patient data [296]. As personalized precision strategies become increasingly intricate in the evolving landscape of oncology, it becomes imperative to integrate real-world data, expansive registries, and comprehensive observational trials [297,298]. This integration is vital for elevating the standard of evidence required for future approvals in the field.

## 5. Glossary

ELISAs—enzyme-linked immunosorbent assays—are an immunologic assay used to detect antibodies, antigens, and proteins. In sandwich ELISA, the plate is first coated with the capture antibody and then the sample is added to the plate, and any antigen in the sample will then bind to the capture antibody [306]. A detection antibody is then added which is labeled with an enzyme that will convert to a color for detection [306]. There are other types of ELISA, including competitive, indirect, and direct, which differ in the methods of detection [306].

Objective/Overall response rates—the proportion of patients in the trial who responded to the agent being measured.

Disease control rates—the percentage of patients whose disease has remained stable or has decreased over a period.

Duration of response—the length of time during which a tumor responds to the treatment without further growth or progression.

Progression-free survival—the length of time during which a person lives without disease progression, such as tumor growth or metastasis.

Median duration of the response—the length of time from initial treatment until half of the patients that responded have experienced disease progression or death.

Disease control rate—the percentage of patients with advance disease whose treatment has led to complete response (disappearance of all signs of neoplasm), partial response, (significant decrease in tumor size), or disease stabilization (no significant increase in tumor size).

Overall survival—the length of time from the start of a treatment regimen until clinical endpoint (i.e., death, disease progression) compared to those in a control group.

## Figures and Tables

**Table 1:** Summary of the next generation sequencing-based oncopanels currently in use in clinical practice and in research. NSCLC: non-small-cell lung cancer.

Panel	# of Genes	Neoplasms	FDA	Manufacturer	Reference
FoundationOne CDx	324	NSCLC, melanoma, breast cancer, colorectal cancer, and ovarian cancer	Yes	Foundation medicine	[47]
Oncomine Dx Target Test	23	Original use: NSCLC Current use: NSCLC, colon cancer, melanoma, gastric and ovarian cancer	Yes	Thermo Fisher scientific	[48]
Omniseq Advance	144	Solid tumors	No	Labcorp oncology	[49]
Omniseq Insight	523	Solid tumors	No	Labcorp oncology	[50]
Cancerplex	435	Solid tumors	No	KEW	[51]

**Table 2:** Programmed death ligand 1 immunohistochemistry assays used to identify programmed death ligand 1-positive cancers. NSCLC, non-small-cell lung cancer.

Stain	Antibody	FDA Approval	Supplier	Reference
22C3	pembrolizumab	Yes for NSCLC	Agilent DAKO	[52,55]
28-8	nivolumab	Yes for NSCLC, Squamous cell carcinoma of the head and neck and melanoma	Agilent DAKO	[52,56]
SP142	atezolizumab	Yes for NSCLC and urothelial carcinoma	Ventana Roche	[52,57]
SP263	durvalumab	Yes for NSCLC	Ventana Roche	[52,58]

## Abbreviations

ALK	anaplastic lymphoma kinase
ATC	anaplastic thyroid cancer
CEA	carcinoembryonic antigen
CRC	colorectal cancer
CTLA-4	cytotoxic T-lymphocyte-associated protein 4
DCs	dendritic cells
DCR	disease control rate
dMMR	mismatch repair-deficient
DOR	duration of response
EGFR	epidermal growth factor receptor
EML4	EMAP-like 4
FDA	United States Food and Drug Administration
FLT3L	FMS-like tyrosine kinase 3 ligand
GDF15	growth differentiation factor 15
HER2	human epidermal growth factor receptor 2
HRR	homologous recombination repair
IHC	immunohistochemistry
InDel	insertions and deletions
LIFl	leukemia inhibitory factor 1
MAPK	Mitogen-activated protein kinase
MCL1	myeloid cell leukemia-1
mDOR	median duration of the response
MDSC	myeloid-derived suppressor cells
MEK1 and MEK2	mitogen-activated protein kinase 1 and 2
MIP	molecular imprinting polymers
MSI-H	metastatic microsatellite instability-high
NGR1	HER3-targeting arm to block neuregulin 1
NGS	Next-generation sequencing
NK	natural killer cell
NPM1	nucleophosmin 1
NSCLC	non-small-cell lung cancer
NTRK	neurotrophic receptor tyrosine kinase
ORR	objective response rate
OS	overall survival
PD-1	programmed death receptor 1
PD-L1	programmed death ligand 1
PFS	progression-free survival
P-GP	P-Glycoprotein
RET	rearranged during transfection
ROS1	receptor tyrosine kinase ROS proto-oncogene 1
TAAs	tumor-associated antigens
TAMs	tumor-ssociated macrophages
TNBC	triple-negative breast cancer
TCGA	The Cancer Genome Atlas Program
TME	tumor microenvironment
TLR	Toll-like receptor
TMB-H	high metastatic tumor mutational burden
